# ATP Synthase and Mitochondrial Bioenergetics Dysfunction in Alzheimer’s Disease

**DOI:** 10.3390/ijms222011185

**Published:** 2021-10-17

**Authors:** Somya Patro, Sujay Ratna, Hianny A. Yamamoto, Andrew T. Ebenezer, Dillon S. Ferguson, Amanpreet Kaur, Brendan C. McIntyre, Ryan Snow, Maria E. Solesio

**Affiliations:** Department of Biology, College of Arts and Sciences, Rutgers University, Camden, NJ 08103, USA; sp2026@scarletmail.rutgers.edu (S.P.); sr1359@scarletmail.rutgers.edu (S.R.); hay6@scarletmail.rutgers.edu (H.A.Y.); ate20@scarletmail.rutgers.edu (A.T.E.); dillonf@princeton.edu (D.S.F.); ak1907@scarletmail.rutgers.edu (A.K.); bcm107@scarletmail.rutgers.edu (B.C.M.); rps109@camden.rutgers.edu (R.S.)

**Keywords:** mitochondria, mitochondrial dysfunction, Alzheimer’s disease, ATPase, ATP synthase, OXPHOS

## Abstract

Alzheimer’s Disease (AD) is the most common neurodegenerative disorder in our society, as the population ages, its incidence is expected to increase in the coming decades. The etiopathology of this disease still remains largely unclear, probably because of the highly complex and multifactorial nature of AD. However, the presence of mitochondrial dysfunction has been broadly described in AD neurons and other cellular populations within the brain, in a wide variety of models and organisms, including post-mortem humans. Mitochondria are complex organelles that play a crucial role in a wide range of cellular processes, including bioenergetics. In fact, in mammals, including humans, the main source of cellular ATP is the oxidative phosphorylation (OXPHOS), a process that occurs in the mitochondrial electron transfer chain (ETC). The last enzyme of the ETC, and therefore the ulterior generator of ATP, is the ATP synthase. Interestingly, in mammalian cells, the ATP synthase can also degrade ATP under certain conditions (ATPase), which further illustrates the crucial role of this enzyme in the regulation of cellular bioenergetics and metabolism. In this collaborative review, we aim to summarize the knowledge of the presence of dysregulated ATP synthase, and of other components of mammalian mitochondrial bioenergetics, as an early event in AD. This dysregulation can act as a trigger of the dysfunction of the organelle, which is a clear component in the etiopathology of AD. Consequently, the pharmacological modulation of the ATP synthase could be a potential strategy to prevent mitochondrial dysfunction in AD.

## 1. Introduction

Alzheimer’s Disease (AD) is the most common neurodegenerative disorder and the leading cause of dementia in the world. In fact, AD affects around 35 million people worldwide and because of the aging global population, the incidence of this pathology is expected to only increase in the coming decades [1]. AD presents in patients with a wide range of symptoms. Some of the earliest symptoms of the disease are memory loss and cognitive decline [2,3]. Over time, AD deleteriously affects other regions of the brain, including the cerebral cortex, which will induce difficulties in speech and reasoning, as well as behavioral alterations in patients [4]. This strongly burdens the health span of patients, and will ultimately cause their death, either directly or through complications associated with the disease.

Interestingly, the main symptoms and the basic etiopathology of AD are mostly common to both the early-onset (before age 60 approximately) and the late-onset (after age 60 approximately) forms of the disease [5]. Furthermore, both forms of AD share some common molecular features, including the presence of specific alleles of apolipoprotein E (ApoE), and decreased levels of the intact postsynaptic protein neurogranin [6]. While early-onset AD is a consequence of mutations in some specific genes, including those coding for presenilin 1 and 2 (*PSEN1* and PSEN2, respectively), and the amyloid precursor protein (APP) [7,8], this type of AD is associated with the familial forms of the disease. Late-onset, which represents approximately 90–95% of cases of AD, is associated with the sporadic forms of the disease [9]. The focus of this review is sporadic AD; therefore, we will refer to this type of disease as just AD.

AD is a multi-causal and highly complex disease, whose exact etiology remains still mostly unknown. Although increased age is the greatest risk factor for the development of the pathology, aging is not considered the exclusive cause of AD, there are a variety of other risk factors including but not limited to family history of AD, gender, ethnicity/race [10]. For example, AD appears to disproportionally affect women. In fact, women are two-thirds more likely to develop AD than men [10,11]. Women live longer than men on average, which elevates their risk for AD. Differences in the presence of AD have also been observed between races and ethnicities. Accordingly, African Americans, when compared to non-Hispanic Caucasians, are twice as likely to develop AD; and Hispanic individuals are between one and a half times more likely to develop AD when compared to non-Hispanic Caucasian Americans [10,11]. The causes for these differences in the incidence of AD in individuals from different genders, races, and ethnicities remain mostly unknown, even if it has been proposed that it could be tied to health disparities, such as those that increase the risk of cardiovascular disease, more than to specific mutations [10,11]. Based on all this, conducting research in the field of AD aimed to advance our knowledge of the etiopathology of the disease is not only a way to improve people’s health spans, but it is also a powerful tool to decrease inequities. 

As previously mentioned, the etiopathology of AD remains unclear. However, a few molecular features of this etiopathology have been identified. For example, the analysis of postmortem brains has identified some common hallmarks of AD, which include but are not limited to the presence of increased amyloid plaques, neurofibrillary tangles (NFTs), synaptic dysfunction, and inflammation [12,13,14,15]. The most common component of the amyloid plaques is the aggregated form of amyloid β (Aβ), which, in AD, accumulates within the brain, both intra- and extra-cellularly. Specifically, intracellular neuronal accumulation of Aβ, as well as in other brain cell types including astrocytes, and microglia; has been broadly demonstrated in both animal models and human samples [12,16,17,18,19,20,21]. Aβ is derived from the breakdown of APP by the enzymes γ-secretase and β-secretase [22], and its neuronal accumulation, increase, and ultimately cell death [23]. The mechanism underlying this rise in cell death is complex and multifactorial. For example, the increased extracellular presence of the aggregated peptide inhibits the communication between different cellular populations within the brain, which contributes to the disruption or the degradation of synapsis, ultimately contributing to increased apoptosis, which is the most common cause of cell death in the brains of AD patients [24]. Moreover, intracellular NFTs are formed by aggregated and phosphorylated tau and α-Synuclein (αSyn) amyloids, among other proteins [25,26]. The synergic and deleterious effects of these two proteins have already been demonstrated [27]. In fact, the presence of insoluble and aggregated tau and αSyn is associated with a decline in neural function [28,29,30]. However, the mechanism underlying these synergic effects is still not well-understood. 

**Figure 1:**
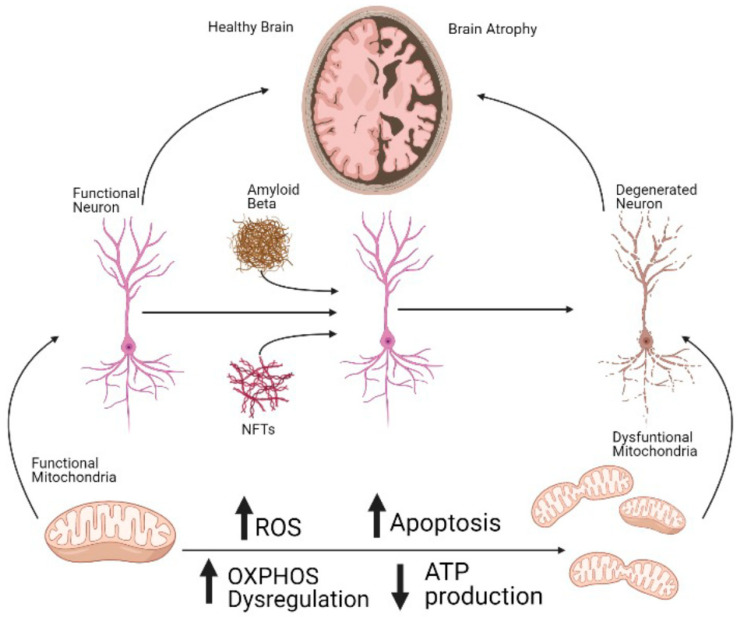
Mitochondrial dysfunction is an early, crucial component of cell death in AD. Dysregulated OXPHOS, including the dysfunction of the ATP synthase, induces decreased ATP production and increased ROS. This will ultimately lead to a rise in the rates of apoptotic cell death not only in neurons but also in other cell types in the central nervous center. The increased death of cells will induce serious damage in the brains of the patients, which correlates with the symptoms of the disease. Interestingly, mitochondrial dysfunction, including dysregulated OXPHOS, is an early event in AD, preceding the accumulation of Aβ and the presence of NTFs. ↑ upregulated; ↓ downregulated.

At the cellular level, the effects of AD in the central nervous system are observed several years before the appearance of the symptoms in the patients [31] (Figure 1). One of the most prevalent of these early features of AD is the presence of mitochondrial dysfunction in diverse cellular types, including astrocytes and neurons [32,33,34]. Interestingly, the dysregulation of the organelle has also been demonstrated in other neurodegenerative disorders and aging [35,36,37,38,39]. In the case of AD, it has been proven that mitochondrial dysfunction precedes the onset of the main symptoms of the disease [40]. Therefore, mitochondrial-addressed strategies have been widely proposed in AD, and some of them have proven to have a positive effect against a part of the cellular effects and symptoms of AD, in diverse cellular and animal models. For instance, the use of antioxidants suppresses the AD-like pathology progression [41]. Furthermore, the inhibition of Drp1, which is the main protein involved in mitochondrial fission [42], decreases dementia, among other molecular hallmarks of AD, including Aβ deposition [43]. Additionally, the use of mitochondrial carbonic anhydrase inhibitors showed a positive effect against Aβ-induced cellular damage [44,45]. 

One of the main components of mitochondrial physiology is the maintenance of an appropriate bioenergetics status. Accordingly, dysregulated bioenergetics is well described in AD [46,47,48,49]. While extra-mitochondrial pathways are also involved in mammalian cellular bioenergetics, the main source of ATP in these organisms is mitochondrial oxidative phosphorylation (OXPHOS) [50]. Dysregulated bioenergetics is especially deleterious for cells in the brain, as neurons and other brain cells are highly metabolically active and consequently, highly energy dependent. For example, one single resting cortical neuron utilizes approximately 4.7 billion ATP/sec [51]. Thus, dysregulated bioenergetics will dramatically affect neuronal populations, the main contributor of increased neuronal cell death in AD. Moreover, the electron transport chain (ETC), the site of OXPHOS, is the main contributor to increased generation of reactive oxygen species (ROS), which is a key contributor towards oxidative stress and another hallmark of AD in neurons, which contributes to the increased cell death present in these cells [52]. 

The mammalian ETC consists of five complexes, along with the transporters ubiquinone and cytochrome C [53]. All five complexes are encoded by both nuclear DNA and mitochondrial DNA (mtDNA), except complex II, which is exclusively encoded by nuclear DNA [53,54,55]. Human complex V, which is also known as the mitochondrial ATP synthase, is ultimately responsible for the production of ATP through the phosphorylation of ADP [56]. Mammalian ATP synthase is composed of two functional domains, F_1_ and F_0_, which are located in the mitochondrial matrix and the inner mitochondrial membrane, respectively [57,58]. The F_0_ subunit is a rotatory proton channel, which also ensures proper anchorage, and stabilization of the F_1_ subunit [59]. While F_0_ is composed of diverse smaller components, the F1 domain is ultimately responsible for the synthesis of ATP [57]. When isolated from mitochondria, and thus uncoupled from the proton gradient, this subunit is known as F_1_-ATPase, as it catalyzes the hydrolysis of ATP [60]. The molecular composition of the F_1_ subunit is α_3_β_3_γδε [61]. Moreover, the oligomycin sensitivity conferral protein (OSCP), is usually also associated with the ATP synthase, which is located in the upper part of the peripheral stalk [62,63]. Interestingly, dimers of the mammalian ATP synthase have also been demonstrated to be components of the mitochondrial permeability transition pore (mPTP) [64], a structure not only crucial for apoptosis, but also for cell survival [65,66].

The dysfunction of diverse complexes of the ETC has been described in a wide variety of pathological scenarios, including in AD. Specifically, the dysregulation and dysfunction of the mitochondrial ATP synthase have been described as one of the cellular hallmarks of this disease, as it is corroborated by a vast bibliography, which we aim to critically review in this manuscript [40,67,68,69,70,71,72,73,74,75,76,77,78,79,80]. It is our humble opinion that further research should be conducted in this field to better validate mitochondrial bioenergetics in general, and more specifically the mammalian ATP synthase, as a pharmacological target to prevent the cellular dysfunctions present in AD. This could significantly contribute towards decreasing cellular damage, including at the mitochondrial level, which is present in neurons and other cellular populations in AD patients, and hopefully, to prevent or decrease the extent of the symptoms of this terrible disease.

## 2. ATP Synthase Dysfunction in AD

As mentioned above, the dysregulation of the F_1_F_0_ ATP synthase has been broadly proposed in different cellular populations of both patients and rodent models of AD [40,67,68,69,70,71,72,73,74,75,76,77,78]. In fact, it has been described that Aβ plaques can directly affect the functioning of complex V [73]. However, many other mechanisms may be involved in the dysregulation of the ATP synthase which is present in AD. For instance, Terni et al. showed that the α subunit of the ATP synthase from the entorhinal cortex of postmortem brains of AD patients has shown to be lipoxidized, due to the increased presence of oxidative stress in these samples, when compared with age-matched samples [71]. Consequently, the same authors have shown that the activity of the ATP synthase was decreased by approximately 30% in the AD samples when compared with control samples. Interestingly, the activity of the complex I of the ETC remained unaffected. These effects were observed even at Braak stages I/II of AD, which are considered early, clinically silent stages of the disease and characterized by tauopathy on the entorhinal and transentorhinal cortices [81]. Interestingly, using self-developed antibodies against insoluble AD brain lesions and human brains which were donated by AD patients, some authors have detected increased accumulation of mitochondrial α subunit of the ATP synthase in the cytosol of AD degenerating neurons, which demonstrates substantial changes in the metabolism of this protein in AD. This accumulation was present even at the early stages of the disease [68]. The same authors showed that this subunit is observed in degenerating neurons either alone or tightly associated with aggregated tau.

The β subunit of the ATP synthase has also been shown to be negatively affected in AD. For example, Tsuji et al., using quantitative proteomic analysis, found that proteins related to the mitochondrial energy metabolism were affected in human brains obtained from AD patients [15]. Specifically, they showed that the β subunit of the ATP synthase was upregulated in these samples, while the presence of the α chain was decreased. The authors suggest that this phenotype could be a crucial component underlying bioenergetics dysregulation in AD. Moreover, using rat cortical primary cultures, a group of authors showed inhibition of the ATP synthase activity and as consequence, increased production of superoxide anion, oxidative damage in complex I of the ETC, mitochondrial membrane depolarization, and ultimately, increased apoptotic cell death in the AD rats, when compared with the control animals. The authors described that this inhibition is a consequence of the dissociation of the anti-apoptotic protein B-cell lymphoma extra-large (Bcl-xL) from the β subunit of the ATP synthase [75]. Moreover, they showed that the mechanisms underlying this dissociation involve the accumulation of cyclin B1 in the areas of the brain affected in AD, due to the inactivation of the anaphase-promoting complex/cyclosome (APC/C)-cadherin 1 (Cdh1), which causes stabilization of cyclin B1, ultimately increasing cell death. Mitochondrial accumulation of B1 binds and activates the cyclin-dependent kinase-1 (Cdk1), forming a B1-Cdk1 complex in the organelle, which phosphorylates Bcl-xL, thus dissociating this protein from the β subunit [75]. Furthermore, Chou et al., using proteomics assays conducted in the cortices of the triple transgenic strain of AD mice (3xTg-AD) and littermate controls, showed an increased presence of the β subunit of the ATP synthase in the mutant mice, compared with the control animals, when they were analyzed using BN/SDS-PAGE 2D-DIGE [82]. The same authors described similar effects in other subunits of the ATP synthase, as well as downregulation of some complexes of the mitochondrial NADH-ubiquinone oxidoreductase. Dysregulation of the β subunit of the ATP synthase, as well as of other subunits of this enzyme and some other enzymes related to OXPHOS, such as the mitochondrial superoxide dismutase 2 (SOD2), was corroborated in the same manuscript, using IEF/SDS-PAGE2D-DIGE. The authors of this study proposed that the upregulation of the proteins that are also dysregulated could be a compensatory mechanism. Differences in the results from this study and the previous one could be related to the specific type of samples that were used (human vs. mice models), as well as to the specific areas in the brain that have been addressed by each study. 

Not only the presence of the different protein components of the ATP synthase but also the levels of the mRNA are affected in AD. For example, it has been shown that the number of mRNA transcripts coding for the ATP synthase β-subunit in nuclear DNA, which is one of the subcomponents of the F_1_, is decreased by 50–60% in the mid-temporal cortex of AD patients, but not in primary motor cortices. Interestingly, mRNAs coding for other components of OXPHOS in both nuclear and mtDNA were also decreased in these samples [83]. However, there is some controversy regarding this last point. In fact, a group of authors, using cerebral cortices and cerebellums from 2-, 5-, and 18-month-old APP transgenic mice (Tg2576), have shown the upregulation of mRNA expression of some mitochondrially encoded genes which are involved in mitochondrial energy metabolism. They have also proven that this upregulation increases as the age of the animals increases. The authors suggest that this could be a compensatory response to the damage induced in mitochondrial energy metabolism by the APP and/or Aβ [84]. This data is corroborated by other studies in which the authors used human brains obtained from AD patients [69,85]. 

As previously mentioned, different studies have shown that the mitochondrial ATP synthase is not only structurally affected in AD, but also that its regulation is compromised in this pathology. For example, using animal models, some authors have demonstrated increased expression of Cyclophilin D (CypD), which is a mitochondrial protein, in aging and AD, where it promotes ATP synthase dysfunction [77]. These results were corroborated and expanded by other authors, who showed that CypD deficiency attenuated ATP synthase dysregulation, restoring bioenergetics, via interaction with the oligomycin-sensitivity conferring protein (OSCP) [76,78]. OSCP is a protein that is located in the peripheral stalk of the ATP synthase, providing structural stability to this enzyme [86]. While the expression of OSCP has been demonstrated to decrease with aging, the interaction between CypD and OSCP increases with aging, most likely due to the increased expression of CypD [77,78]. Interestingly, the decreased presence of OSCP in AD seems to be independent of the expression of the different subunits of the ATP synthase, as demonstrated using 5XFAD mice and mitochondria isolated from synaptic and non-synaptic cells [76]. Specifically, the CypD/OSCP interaction has been demonstrated to lower OXPHOS activity by uncoupling the F_1_F_0_ subunit of the ATP synthase, which increases oxidative stress via increased superoxide production, and ultimately triggers the formation and the opening of the mPTP in the cellular populations in the brains of patients of AD [76,77]. These effects might be induced by the direct interaction between Aβ and OSCP, which has been demonstrated in models of AD [76,78].

Moreover, the cellular signaling system involved in sensing glucose, and therefore, the activation of the ATP synthase, has also been demonstrated to be dysregulated in AD. For example, suppressed *O*-GlcNAc (glycosylation with O-linked β-*N*-acetylglucosamine) has been proven in diverse models of AD [74]. Glycosylation of the α subunit from the ATP synthase by *O*-GlcNAc is a signal for the enzyme to activate ATP production [74]. The same authors demonstrated that the mechanism underlying this process is the direct binding between Aβ and the ATP synthase, which inhibits the direct interaction between the α subunit of the enzyme and the *O*-GlcNAc transferase. Additionally, they have also proven that the treatment with pharmacological inhibitors of the *O*-GlcNAcase was able to rescue the impairment of ATP production. Moreover, the deleterious effects of decreased *O*-GlcNAc on tau pathology have also been corroborated using cellular models of AD [87]. *O*-GlcNAc is a post-translational modification that adds an N-acetylglucosamine moiety to serine or threonine residues in target proteins that contain an *O*-glycosidic bond, has been demonstrated to be a glucose sensor [88]. The mechanism underlying this sensing system involves uridine diphosphate-*N*-acetylglucosamine, which is a natural source of *O*-GlcNAc, and it is produced from extracellular glucose by the hexosamine biosynthetic pathway [89].

## 3. Dysregulation of Other Complexes of the ETC in AD

As previously mentioned, ETC is formed by five complexes, and the function of all of them is closely inter-connected. Consequently, dysregulation of complexes other than the ATP synthase has also been described in AD. In fact, decreased expression of genes coding for OXPHOS, especially of those involved in complex I, has been demonstrated in AD. Furthermore, the synergic effects of aggregated Aβ and tau to reduce overall mitochondrial function have also been proven by Rhein et al. [90]. The authors of this study demonstrated that tau is specifically responsible for the early bioenergetic dysfunction, present in Braak stage I/II. This increases the vulnerability of mitochondrial proteins to damage by Aβ in the late stages of AD. Lastly, Aβ plaques have also been shown to be per se an inhibitor of complex IV via decreased ability for affected neurons to depolarize [91]. 

The literature is rich with examples that show the presence of defects in the diverse complexes of the ETC in AD. For example, some authors have shown that tau tangles, which are formed during AD, are associated with a 31% decrease in the activity of complex I [92,93]. Moreover, other authors have demonstrated that approximately one-third of the proteins associated with cytochrome C oxidase (COX), which is complex IV of the ETC; as well as the levels of NADH, which is a substrate for complex I, have both been shown to be reduced due to the accumulation of aggregated Aβ in AD [94,95,96,97]. In fact, the joint dysregulation of complex II and IV in AD has been proven [90]. Additionally, Adav et al. showed in a quantitative proteomic study which was conducted comparing temporal cortices which were obtained from AD patients and age-matched controls, the deleterious effects that the progression of AD plays in the dysregulation of mitochondrial bioenergetics [98]. The authors of this in-depth study showed results spanning across ETC complexes I through V. Although all the complexes were affected, the authors found the degree of this affection to be greater in the subunits of complex I. Interestingly, the effects of the dysregulation of the coupling of complex I with the rest of the components of the ETC have also been shown to have a deleterious effect on the synthesis of ATP which is present in AD [93]. Accordingly, complex I deficiencies have been shown to correlate with complex V dysfunction, with the consequent decreased production of ATP and increased generation of ROS [92]. Interestingly, increased ROS has been demonstrated not only in AD but also in general aging [35]. In addition, Reddy et al., using postmortem brains which were obtained from AD patients, showed downregulation of mRNA coding for complex I of the ETC in these patients. However, in accordance with other previously mentioned studies [84], complexes III and IV were upregulated in these samples, when compared with the samples obtained from healthy individuals. The data also showed differential expression of ATPase δ-subunit in AD patients. These changes were present even in brains obtained from early-stage AD patients. The authors, however, recognize a large intra-personal variation in the data between the different individuals and suggest conducting further studies to increase the sample size [69]. Lastly, it has been demonstrated that the dysregulation of COX, which leads to a decline in ATP production as previously mentioned, also leads to decreased mitochondrial membrane potential, which will, ultimately, increase apoptotic cell death [99,100]. The effects of AD in COX have been described within the neurons in the hippocampus, which is one of the areas of the brain where a higher number of senile plaques and NFTs accumulate [101,102]. In fact, it has been demonstrated that there is a 35–40% decrease in the activity of COX in hippocampi from AD patients when compared with age-matched individuals [67]. These results were corroborated in animal models, where decreased COX activity because of the treatment with the Aβ_25-35_ peptide was found in rats, as well as in the hippocampi of mice as a consequence of the increased presence of Aβ plaques [15,91,103]. Accordingly, other authors also showed decreased levels of the mitochondrially-coded COX subunits I and III in the association cortex from AD patients, again when compared with age-matched controls [69,85,104]. 

**Figure 2:**
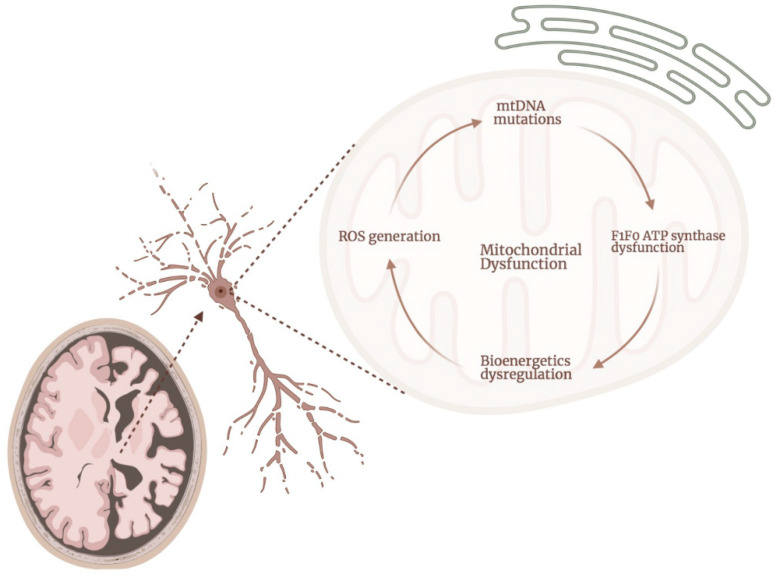
Mitochondrial dysfunction is present at different levels in mitochondria, in a deleterious cycle which increases the damage to the organelle and the cells in AD. Dysregulation of the mitochondrial ATP synthase is present in neurons and other cellular populations in the brains of AD patients. This dysregulation will further contribute towards bioenergetics dysfunction and the consequent increased production of ROS. That rise in the levels of ROS is one of the main causes underlying the damage present in AD in the mtDNA, which will further affect the expression and the activity of the ATP synthase, starting the deleterious cycle again.

The close and deleterious interconnection between increased production of ROS and mitochondrial dysfunction, including bioenergetics dysregulation, has been broadly demonstrated in both human and animal models of AD [90,105,106,107,108,109]. Specifically, increased oxidative stress negatively affects the integrity of mtDNA (including base mispairing, random point mutations, and deletions), and the status of protein homeostasis, damaging the complexes of the ETC and dysregulating mitochondrial bioenergetics, in a vicious cycle which ultimately induces increased apoptotic cell death [110,111,112,113] (Figure 2). For example, various researchers have hypothesized that the mechanism affecting COX in AD involves ROS. Specifically, it has been proven that this complex is very sensitive to changes in the fluidity and the composition of the membrane lipids. This is because some of these lipids, including cardiolipin, phosphatidylcholine, and phosphatidylethanolamine, are essential for the functioning of this subunit [114]. Under situations where a rise in ROS generation is present, these lipids can be peroxidated, which will ultimately affect the activity of complex IV [115]. Accordingly, decreased activity of complex IV and increased lipid peroxidation has been found in various samples, including brains obtained from patients of AD [116,117,118]. At the same time, this affection of complex IV increases the generation of superoxide by complexes I–III [119]. Interestingly, Aβ aggregates also have been shown to directly interact with the lipidic membranes [120]. In fact, Aβ has been demonstrated to induce intracellular accumulation of hydrogen peroxide and/or lipid peroxide in models of AD [84,121], which prolonged presence in neurons and other cellular populations in the brain will ultimately result in cellular oxidative damage. Interestingly, Kawamoto et al. demonstrated that the massive increase in the production of ROS that is produced in AD is not only present in neurons and brain cellular populations, but also in other cells, including platelets and erythrocytes [70]. Lastly, Korolainen et al. found decreased levels of oxidation in proteins from brains obtained post-mortem from AD patients when compared with control, aged-matched brains [122]. This for example was the case with the mitochondrial glutamate dehydrogenase. The authors of this study proposed that these findings do not contradict the bibliography suggesting a crucial role for ROS in the cellular damage observed in AD. They, in fact, suggest that their findings could be a co-existing compensatory post-transcriptional response [122]. Interestingly, in the same study, the authors show increased expression of the β subunit of the ATP synthase in the AD samples, when compared with control samples. However, no changes in the oxidative status of this protein were described. 

Some pharmacological approaches have been proposed to counteract or to prevent bioenergetics dysfunction in AD, with the hope of decreasing the cellular damage that is present in this condition. For instance, some studies conducted in various models have shown that decreased levels of complex IV activity, which is closely related to the ATP synthase, lead to reduced NADH oxidation [123]. Based on these findings, some authors have proposed the oral treatment with NADH, or even with the dehydrogenated form of this molecule (NAD^+^), as a pharmacological tool in AD [124,125]. Additionally, the use of small molecules to modulate complex I has also demonstrated protective effects against cognitive decline in various mice models, extending their lifespan [126,127]. Moreover, some authors have proposed to mitigate neuroinflammation as a pharmacological tool in AD [128]. Interestingly, COX plays a crucial role in the regulation of neuroinflammation in mammals [129]. Furthermore, the regulation of the cellular secretion pathways and exocytosis, which are closely related to the bioenergetic status of the cells, have also been proposed as a pharmacological strategy in AD [130]. Lastly, using mice models of AD (APP/SEN1) where increased production of ROS has been demonstrated, Dixit et al. showed that while the deficiency of ascorbate, which is a well-known antioxidant, exacerbates mitochondrial oxidative stress as expected, the supplementation with this drug, not only mitigates the generation of ROS, but it also prevents mitochondrial membrane depolarization and, therefore, bioenergetics dysregulation [131].

## 4. Mitochondrial Calcium Homeostasis

Mitochondrial calcium homeostasis is closely related to the bioenergetic status of the cells, as well as to the opening of the mPTP [132], which is a crucial structure in cell fate, including apoptosis. The concentration of resting mitochondrial calcium, which is usually higher than the cytoplasmic concentration of the cation, has been defined as approximately 100–200 nM, even if it considerably increases when calcium signals are activated [133,134,135]. Intramitochondrial calcium is crucial in the production of ATP by activating the dehydrogenase enzymes within the organelle, including those involved in the tricarboxylic cycle, which leads to increased levels of NADH and ATP generation [136]. While mitochondrial-free calcium homeostasis still remains poorly understood, it has been proposed that inorganic polyphosphate (polyP) could play a key role in this process [137,138]. Interestingly, polyP has also been demonstrated to be crucial in mammalian bioenergetics [47], including the regulation of the mPTP [65,139], as well as in the physiology of the endoplasmic reticulum (ER) [140]. Furthermore, this polymer has also been proposed to have a protective role in neurodegenerative disorders, including AD [48,49]. 

Dysregulated mitochondrial calcium homeostasis has been broadly described in AD. For example, in a study conducted using cytoplasmic hybrid (cybrid) cell lines obtained from AD patients, Sheehan et al. showed decreased ability to buffer increasing cytoplasmic calcium concentrations by mitochondria in these samples, compared with the cells cybrids which were prepared from control patients. This dysregulated calcium homeostasis will ultimately induce mitochondrial dysfunction, increased ROS production, mtDNA mutations, and apoptosis [141]. In fact, as previously mentioned, mitochondria have been shown to be a direct site for the accumulation of aggregated Aβ in the neurons of both animal models of AD and samples obtained from patients of the disease. One of the mechanisms that can explain the cytotoxicity of the peptide involves the mechanisms of the calcium transfer from ER to mitochondria, which has been demonstrated in various models of AD [142,143,144,145]. Specifically, Boyman et al. showed that the content of the mitochondrial calcium uniporter (MCU), a structure that allows calcium to flow from the ER to mitochondria [146], significantly increases in fibroblasts obtained from AD patients, when compared with healthy age-matched individuals [147]. The same authors demonstrated that this increased presence of MCU further causes mitochondrial calcium levels in the organelle to increase. In fact, the pharmacological blockage of MCU has been proposed as a therapeutic strategy against AD, to prevent mitochondrial calcium uptake from the ER. For example, the use of Ruthenium 360, which is a well-known inhibitor of MCU, prevented Aβ-induced mitochondrial calcium overloading [143].

The interaction between mitochondria and ER in mammalian cells is mediated by the mitochondria-associated ER membranes (MAMs) [148]. MAMs play a crucial role not only in calcium homeostasis but also in the regulation of lipid synthesis, mitochondrial dynamics, energy metabolism, cell survival, and apoptotic signaling [142,145]. Interestingly, using mammalian cells, Area-Gomez et al. demonstrated that in AD models there is an upregulation of MAM activity, which elevates the crosstalk between mitochondria and ER, deleteriously affecting mitochondrial calcium homeostasis [149]. Moreover, the upregulation of MAM-associated proteins, which increases the number of ER-mitochondria contact points, as well as the MAM-associated protein expression in the primary hippocampal neurons extracted from postmortem human samples from AD patients and from transgenic models of the diseases, has also been demonstrated [142,145]. These effects were observed even before the presence and development of Aβ plaques and NTFs. Lastly, it is of note that the effects of dysfunctional MAMs are not only present in neurons, but also in other cellular populations where mitochondrial dysfunction has been described in AD, such as the case of human fibroblasts. In fact, Supnet et al. have found that fibroblasts obtained from AD patients present a significant increase in cytosolic calcium levels when compared to healthy age-matched controls after treatment with thapsigargin [150], which is a drug that allows for the release of calcium from the ER stores, which induces mitochondrial calcium accumulation [151]. The use of this drug has been shown to induce the opening of the mPTP, depolarize mitochondria, and ultimately activate apoptosis [152]. In another study, which was conducted using fibroblasts from AD patients, Gibson et al. showed that after the treatment with thapsigargin, the mitochondrial length, membrane potential, and buffering capacity were significantly reduced in the samples obtained from the AD patients, when compared to fibroblasts obtained from young and healthy individuals [153].

As previously mentioned, the accumulation of calcium in mitochondria triggers the formation and the opening of the mPTP [132], which is a structure closely related to apoptosis. Therefore, dysregulated mPTP has also been demonstrated in AD. For example, using human fibroblasts from AD patients, some patients have shown that the opening of the mPTP is a crucial contributor towards increased mitochondrial dysfunction in these samples when compared with fibroblasts isolated from age-matched control individuals [147]. Moreover, Perez et al. demonstrated the full and permanent opening of the mPTP in fibroblasts obtained from AD patients when compared with fibroblasts obtained from healthy, age-matched individuals [147]. The same authors, propose that the opening of the mPTP is a pathological event, triggering the apoptotic signaling cascade since it allows for calcium efflux from the inner mitochondrial membrane, which ultimately increases the mitochondrial levels of calcium, as cytosolic calcium levels continue to elevate.

## 5. Conclusions

While the presence of bioenergetics dysfunction, including the dysregulation of the mammalian ATP synthase, is clear in AD, the mechanisms underlying this dysfunction remain still mostly unclear. This might be due to the high complexity of mitochondrial bioenergetics. In fact, mitochondrial bioenergetics is crucial not only for the maintenance of appropriate bioenergetics through the production of ATP, but it is also pivotal in the regulation of some other mitochondrial and cellular processes which are, consequently, affected in AD. This is the case of the oxidative status of the cells and the metabolism of calcium, among others. Dysregulation of these processes can further increase the degree of mitochondrial dysfunction and of the cellular damage induced by dysfunctional bioenergetics, creating a vicious and deleterious cycle, which will ultimately drive the cell to apoptosis.

It is our humble opinion that further research should be conducted in this field to clarify the exact mechanisms underlying mitochondrial bioenergetics dysregulation in AD, which, as previously mentioned, has been proven to be an early event in this pathology. While some pharmacological strategies have already been proposed and tested in different models of the disease, there is still much work left to do to cure AD. Just by increasing our knowledge about these mechanisms, we will be able to validate the mammalian ATP synthase as a pharmacological target in AD and subsequently to search for therapeutic tools that will allow us to prevent mitochondrial dysfunction and, hopefully, to ameliorate the symptoms or to prevent or delay the progression of the disease.

## References

[1] Association A.s. (2021). 2021 Alzheimer’s disease facts and figures. Alzheimers Dement..

[2] Ten Kate M., Dicks E., Visser P.J., van der Flier W.M., Teunissen C.E., Barkhof F., Scheltens P., Tijms B.M., Alzheimer’s Disease Neuroimaging Initiative (2018). Atrophy subtypes in prodromal Alzheimer’s disease are associated with cognitive decline. Brain.

[3] James B.D., Wilson R.S., Capuano A.W., Boyle P.A., Shah R.C., Lamar M., Ely E.W., Bennett D.A., Schneider J.A. (2019). Hospitalization, Alzheimer’s Disease and Related Neuropathologies, and Cognitive Decline. Ann. Neurol..

[4] Ahmed S., Haigh A.M., de Jager C.A., Garrard P. (2013). Connected speech as a marker of disease progression in autopsy-proven Alzheimer’s disease. Brain.

[5] Duara R., Lopez-Alberola R.F., Barker W.W., Loewenstein D.A., Zatinsky M., Eisdorfer C.E., Weinberg G.B. (1993). A comparison of familial and sporadic Alzheimer’s disease. Neurology.

[6] Kvartsberg H., Lashley T., Murray C.E., Brinkmalm G., Cullen N.C., Hoglund K., Zetterberg H., Blennow K., Portelius E. (2019). The intact postsynaptic protein neurogranin is reduced in brain tissue from patients with familial and sporadic Alzheimer’s disease. Acta Neuropathol..

[7] Hsu S., Pimenova A.A., Hayes K., Villa J.A., Rosene M.J., Jere M., Goate A.M., Karch C.M. (2020). Systematic validation of variants of unknown significance in APP, PSEN1 and PSEN2. Neurobiol. Dis..

[8] Wisniewski T., Dowjat W.K., Permanne B., Palha J., Kumar A., Gallo G., Frangione B. (1997). Presenilin-1 is associated with Alzheimer’s disease amyloid. Am. J. Pathol..

[9] Harman D. (2006). Alzheimer’s disease pathogenesis: Role of aging. Ann. N. Y. Acad. Sci..

[10] Matthews K.A., Xu W., Gaglioti A.H., Holt J.B., Croft J.B., Mack D., McGuire L.C. (2019). Racial and ethnic estimates of Alzheimer’s disease and related dementias in the United States (2015–2060) in adults aged >/=65 years. Alzheimers Dement..

[11] Alzheimer’s Association (2020). 2020 Alzheimer’s disease facts and figures. Alzheimers Dement..

[12] Oddo S., Caccamo A., Shepherd J.D., Murphy M.P., Golde T.E., Kayed R., Metherate R., Mattson M.P., Akbari Y., LaFerla F.M. (2003). Triple-transgenic model of Alzheimer’s disease with plaques and tangles: Intracellular Abeta and synaptic dysfunction. Neuron.

[13] Malpetti M., Kievit R.A., Passamonti L., Jones P.S., Tsvetanov K.A., Rittman T., Mak E., Nicastro N., Bevan-Jones W.R., Su L. (2020). Microglial activation and tau burden predict cognitive decline in Alzheimer’s disease. Brain.

[14] Murphy M.P., LeVine H. (2010). Alzheimer’s disease and the amyloid-beta peptide. J. Alzheimers Dis..

[15] Tsuji T., Shiozaki A., Kohno R., Yoshizato K., Shimohama S. (2002). Proteomic profiling and neurodegeneration in Alzheimer’s disease. Neurochem. Res..

[16] Nagele R.G., D’Andrea M.R., Lee H., Venkataraman V., Wang H.Y. (2003). Astrocytes accumulate A beta 42 and give rise to astrocytic amyloid plaques in Alzheimer disease brains. Brain Res..

[17] Wirths O., Multhaup G., Czech C., Blanchard V., Moussaoui S., Tremp G., Pradier L., Beyreuther K., Bayer T.A. (2001). Intraneuronal Abeta accumulation precedes plaque formation in beta-amyloid precursor protein and presenilin-1 double-transgenic mice. Neurosci. Lett..

[18] Welikovitch L.A., Do Carmo S., Magloczky Z., Malcolm J.C., Loke J., Klein W.L., Freund T., Cuello A.C. (2020). Early intraneuronal amyloid triggers neuron-derived inflammatory signaling in APP transgenic rats and human brain. Proc. Natl. Acad. Sci. USA.

[19] LaFerla F.M., Green K.N., Oddo S. (2007). Intracellular amyloid-beta in Alzheimer’s disease. Nat. Rev. Neurosci..

[20] Zotova E., Holmes C., Johnston D., Neal J.W., Nicoll J.A., Boche D. (2011). Microglial alterations in human Alzheimer’s disease following Abeta42 immunization. Neuropathol. Appl. Neurobiol..

[21] Liao M.C., Muratore C.R., Gierahn T.M., Sullivan S.E., Srikanth P., De Jager P.L., Love J.C., Young-Pearse T.L. (2016). Single-Cell Detection of Secreted Abeta and sAPPalpha from Human IPSC-Derived Neurons and Astrocytes. J. Neurosci..

[22] Vassar R., Bennett B.D., Babu-Khan S., Kahn S., Mendiaz E.A., Denis P., Teplow D.B., Ross S., Amarante P., Loeloff R. (1999). Beta-secretase cleavage of Alzheimer’s amyloid precursor protein by the transmembrane aspartic protease BACE. Science.

[23] Choi H., Kim C., Song H., Cha M.Y., Cho H.J., Son S.M., Kim H.J., Mook-Jung I. (2019). Amyloid beta-induced elevation of O-GlcNAcylated c-Fos promotes neuronal cell death. Aging Cell.

[24] Shimohama S. (2000). Apoptosis in Alzheimer’s disease—An update. Apoptosis.

[25] Blennow K., Vanmechelen E., Hampel H. (2001). CSF total tau, Abeta42 and phosphorylated tau protein as biomarkers for Alzheimer’s disease. Mol. Neurobiol..

[26] Serrano-Pozo A., Frosch M.P., Masliah E., Hyman B.T. (2011). Neuropathological alterations in Alzheimer disease. Cold Spring Harb. Perspect. Med..

[27] Lu J., Zhang S., Ma X., Jia C., Liu Z., Huang C., Liu C., Li D. (2020). Structural basis of the interplay between alpha-synuclein and Tau in regulating pathological amyloid aggregation. J. Biol. Chem..

[28] Iba M., Guo J.L., McBride J.D., Zhang B., Trojanowski J.Q., Lee V.M. (2013). Synthetic tau fibrils mediate transmission of neurofibrillary tangles in a transgenic mouse model of Alzheimer’s-like tauopathy. J. Neurosci..

[29] Drummond E., Pires G., MacMurray C., Askenazi M., Nayak S., Bourdon M., Safar J., Ueberheide B., Wisniewski T. (2020). Phosphorylated tau interactome in the human Alzheimer’s disease brain. Brain.

[30] Estus S., Tucker H.M., van Rooyen C., Wright S., Brigham E.F., Wogulis M., Rydel R.E. (1997). Aggregated amyloid-beta protein induces cortical neuronal apoptosis and concomitant “apoptotic” pattern of gene induction. J. Neurosci..

[31] De Strooper B., Karran E. (2016). The Cellular Phase of Alzheimer’s Disease. Cell.

[32] Abramov A.Y., Canevari L., Duchen M.R. (2004). Beta-amyloid peptides induce mitochondrial dysfunction and oxidative stress in astrocytes and death of neurons through activation of NADPH oxidase. J. Neurosci..

[33] Bell S.M., Barnes K., De Marco M., Shaw P.J., Ferraiuolo L., Blackburn D.J., Venneri A., Mortiboys H. (2021). Mitochondrial Dysfunction in Alzheimer’s Disease: A Biomarker of the Future?. Biomedicines.

[34] Onyango I.G., Dennis J., Khan S.M. (2016). Mitochondrial Dysfunction in Alzheimer’s Disease and the Rationale for Bioenergetics Based Therapies. Aging Dis..

[35] Baltanas A., Solesio M.E., Zalba G., Galindo M.F., Fortuno A., Jordan J. (2013). The senescence-accelerated mouse prone-8 (SAM-P8) oxidative stress is associated with upregulation of renal NADPH oxidase system. J. Physiol. Biochem..

[36] Galindo M.F., Solesio M.E., Atienzar-Aroca S., Zamora M.J., Jordan Bueso J. (2012). Mitochondrial dynamics and mitophagy in the 6-hydroxydopamine preclinical model of Parkinson’s disease. Parkinsons Dis..

[37] Solesio M.E., Prime T.A., Logan A., Murphy M.P., Del Mar Arroyo-Jimenez M., Jordan J., Galindo M.F. (2013). The mitochondria-targeted anti-oxidant MitoQ reduces aspects of mitochondrial fission in the 6-OHDA cell model of Parkinson’s disease. Biochim. Biophys. Acta.

[38] Solesio M.E., Saez-Atienzar S., Jordan J., Galindo M.F. (2012). Characterization of mitophagy in the 6-hydoxydopamine Parkinson’s disease model. Toxicol. Sci..

[39] Solesio M.E., Saez-Atienzar S., Jordan J., Galindo M.F. (2013). 3-Nitropropionic acid induces autophagy by forming mitochondrial permeability transition pores rather than activating the mitochondrial fission pathway. Br. J. Pharmacol..

[40] Lunnon K., Keohane A., Pidsley R., Newhouse S., Riddoch-Contreras J., Thubron E.B., Devall M., Soininen H., Kloszewska I., Mecocci P. (2017). Mitochondrial genes are altered in blood early in Alzheimer’s disease. Neurobiol. Aging.

[41] Stefanova N.A., Ershov N.I., Kolosova N.G. (2019). Suppression of Alzheimer’s Disease-Like Pathology Progression by Mitochondria-Targeted Antioxidant SkQ1: A Transcriptome Profiling Study. Oxid. Med. Cell. Longev..

[42] Taguchi N., Ishihara N., Jofuku A., Oka T., Mihara K. (2007). Mitotic phosphorylation of dynamin-related GTPase Drp1 participates in mitochondrial fission. J. Biol. Chem..

[43] Baek S.H., Park S.J., Jeong J.I., Kim S.H., Han J., Kyung J.W., Baik S.H., Choi Y., Choi B.Y., Park J.S. (2017). Inhibition of Drp1 Ameliorates Synaptic Depression, Abeta Deposition, and Cognitive Impairment in an Alzheimer’s Disease Model. J. Neurosci..

[44] Solesio M.E., Peixoto P.M., Debure L., Madamba S.M., de Leon M.J., Wisniewski T., Pavlov E.V., Fossati S. (2018). Carbonic anhydrase inhibition selectively prevents amyloid beta neurovascular mitochondrial toxicity. Aging Cell.

[45] Fossati S., Giannoni P., Solesio M.E., Cocklin S.L., Cabrera E., Ghiso J., Rostagno A. (2016). The carbonic anhydrase inhibitor methazolamide prevents amyloid beta-induced mitochondrial dysfunction and caspase activation protecting neuronal and glial cells in vitro and in the mouse brain. Neurobiol. Dis..

[46] Yao J., Irwin R.W., Zhao L., Nilsen J., Hamilton R.T., Brinton R.D. (2009). Mitochondrial bioenergetic deficit precedes Alzheimer’s pathology in female mouse model of Alzheimer’s disease. Proc. Natl. Acad. Sci. USA.

[47] Solesio M.E., Xie L., McIntyre B., Ellenberger M., Mitaishvili E., Bhadra-Lobo S., Bettcher L.F., Bazil J.N., Raftery D., Jakob U. (2021). Depletion of mitochondrial inorganic polyphosphate (polyP) in mammalian cells causes metabolic shift from oxidative phosphorylation to glycolysis. Biochem. J..

[48] McIntyre B., Solesio M.E. (2021). Mitochondrial inorganic polyphosphate (polyP): The missing link of mammalian bioenergetics. Neural. Regen. Res..

[49] Borden E.A., Furey M., Gattone N.J., Hambardikar V.D., Liang X.H., Scoma E.R., Abou Samra A., LR D.G., Dennis D.J., Fricker D. (2021). Is there a link between inorganic polyphosphate (polyP), mitochondria, and neurodegeneration?. Pharmacol. Res..

[50] Krebs H.A., Ruffo A., Johnson M., Eggleston L.V., Hems R. (1953). Oxidative phosphorylation. Biochem. J..

[51] Zhu X.H., Qiao H., Du F., Xiong Q., Liu X., Zhang X., Ugurbil K., Chen W. (2012). Quantitative imaging of energy expenditure in human brain. Neuroimage.

[52] Markesbery W.R. (1999). The role of oxidative stress in Alzheimer disease. Arch. Neurol..

[53] Tzagoloff A., Myers A.M. (1986). Genetics of mitochondrial biogenesis. Annu. Rev. Biochem..

[54] Lazarou M., Smith S.M., Thorburn D.R., Ryan M.T., McKenzie M. (2009). Assembly of nuclear DNA-encoded subunits into mitochondrial complex IV, and their preferential integration into supercomplex forms in patient mitochondria. FEBS J..

[55] Lemarie A., Grimm S. (2011). Mitochondrial respiratory chain complexes: Apoptosis sensors mutated in cancer?. Oncogene.

[56] Jonckheere A.I., Smeitink J.A., Rodenburg R.J. (2012). Mitochondrial ATP synthase: Architecture, function and pathology. J. Inherit. Metab. Dis..

[57] Menz R.I., Walker J.E., Leslie A.G. (2001). Structure of bovine mitochondrial F(1)-ATPase with nucleotide bound to all three catalytic sites: Implications for the mechanism of rotary catalysis. Cell.

[58] Pinke G., Zhou L., Sazanov L.A. (2020). Cryo-EM structure of the entire mammalian F-type ATP synthase. Nat. Struct. Mol. Biol..

[59] Suzuki T., Ueno H., Mitome N., Suzuki J., Yoshida M. (2002). F(0) of ATP synthase is a rotary proton channel. Obligatory coupling of proton translocation with rotation of c-subunit ring. J. Biol. Chem..

[60] Dittrich M., Hayashi S., Schulten K. (2003). On the mechanism of ATP hydrolysis in F1-ATPase. Biophys. J..

[61] Xu T., Pagadala V., Mueller D.M. (2015). Understanding structure, function, and mutations in the mitochondrial ATP synthase. Microb. Cell.

[62] Antoniel M., Giorgio V., Fogolari F., Glick G.D., Bernardi P., Lippe G. (2014). The oligomycin-sensitivity conferring protein of mitochondrial ATP synthase: Emerging new roles in mitochondrial pathophysiology. Int. J. Mol. Sci..

[63] Devenish R.J., Prescott M., Boyle G.M., Nagley P. (2000). The oligomycin axis of mitochondrial ATP synthase: OSCP and the proton channel. J. Bioenerg. Biomembr..

[64] Giorgio V., von Stockum S., Antoniel M., Fabbro A., Fogolari F., Forte M., Glick G.D., Petronilli V., Zoratti M., Szabo I. (2013). Dimers of mitochondrial ATP synthase form the permeability transition pore. Proc. Natl. Acad. Sci. USA.

[65] Amodeo G.F., Solesio M.E., Pavlov E.V. (2017). From ATP synthase dimers to C-ring conformational changes: Unified model of the mitochondrial permeability transition pore. Cell Death Dis..

[66] Neginskaya M.A., Solesio M.E., Berezhnaya E.V., Amodeo G.F., Mnatsakanyan N., Jonas E.A., Pavlov E.V. (2019). ATP Synthase C-Subunit-Deficient Mitochondria Have a Small Cyclosporine A-Sensitive Channel, but Lack the Permeability Transition Pore. Cell Rep..

[67] Bosetti F., Brizzi F., Barogi S., Mancuso M., Siciliano G., Tendi E.A., Murri L., Rapoport S.I., Solaini G. (2002). Cytochrome c oxidase and mitochondrial F1F0-ATPase (ATP synthase) activities in platelets and brain from patients with Alzheimer’s disease. Neurobiol. Aging.

[68] Sergeant N., Wattez A., Galvan-valencia M., Ghestem A., David J.P., Lemoine J., Sautiere P.E., Dachary J., Mazat J.P., Michalski J.C. (2003). Association of ATP synthase alpha-chain with neurofibrillary degeneration in Alzheimer’s disease. Neuroscience.

[69] Manczak M., Park B.S., Jung Y., Reddy P.H. (2004). Differential expression of oxidative phosphorylation genes in patients with Alzheimer’s disease: Implications for early mitochondrial dysfunction and oxidative damage. Neuromol. Med..

[70] Kawamoto E.M., Munhoz C.D., Glezer I., Bahia V.S., Caramelli P., Nitrini R., Gorjao R., Curi R., Scavone C., Marcourakis T. (2005). Oxidative state in platelets and erythrocytes in aging and Alzheimer’s disease. Neurobiol. Aging.

[71] Terni B., Boada J., Portero-Otin M., Pamplona R., Ferrer I. (2010). Mitochondrial ATP-synthase in the entorhinal cortex is a target of oxidative stress at stages I/II of Alzheimer’s disease pathology. Brain Pathol..

[72] Amadoro G., Corsetti V., Atlante A., Florenzano F., Capsoni S., Bussani R., Mercanti D., Calissano P. (2012). Interaction between NH(2)-tau fragment and Abeta in Alzheimer’s disease mitochondria contributes to the synaptic deterioration. Neurobiol. Aging.

[73] Xing S.L., Chen B., Shen D.Z., Zhu C.Q. (2012). beta-amyloid peptide binds and regulates ectopic ATP synthase alpha-chain on neural surface. Int. J. Neurosci..

[74] Cha M.Y., Cho H.J., Kim C., Jung Y.O., Kang M.J., Murray M.E., Hong H.S., Choi Y.J., Choi H., Kim D.K. (2015). Mitochondrial ATP synthase activity is impaired by suppressed O-GlcNAcylation in Alzheimer’s disease. Hum. Mol. Genet..

[75] Veas-Perez de Tudela M., Delgado-Esteban M., Maestre C., Bobo-Jimenez V., Jimenez-Blasco D., Vecino R., Bolanos J.P., Almeida A. (2015). Regulation of Bcl-xL-ATP Synthase Interaction by Mitochondrial Cyclin B1-Cyclin-Dependent Kinase-1 Determines Neuronal Survival. J. Neurosci..

[76] Beck S.J., Guo L., Phensy A., Tian J., Wang L., Tandon N., Gauba E., Lu L., Pascual J.M., Kroener S. (2016). Deregulation of mitochondrial F1FO-ATP synthase via OSCP in Alzheimer’s disease. Nat. Commun..

[77] Gauba E., Guo L., Du H. (2017). Cyclophilin D Promotes Brain Mitochondrial F1FO ATP Synthase Dysfunction in Aging Mice. J. Alzheimers Dis..

[78] Gauba E., Chen H., Guo L., Du H. (2019). Cyclophilin D deficiency attenuates mitochondrial F1Fo ATP synthase dysfunction via OSCP in Alzheimer’s disease. Neurobiol. Dis..

[79] Galber C., Carissimi S., Baracca A., Giorgio V. (2021). The ATP Synthase Deficiency in Human Diseases. Life.

[80] Ebanks B., Ingram T.L., Chakrabarti L. (2020). ATP synthase and Alzheimer’s disease: Putting a spin on the mitochondrial hypothesis. Aging.

[81] Braak H., Alafuzoff I., Arzberger T., Kretzschmar H., Del Tredici K. (2006). Staging of Alzheimer disease-associated neurofibrillary pathology using paraffin sections and immunocytochemistry. Acta Neuropathol..

[82] Chou J.L., Shenoy D.V., Thomas N., Choudhary P.K., Laferla F.M., Goodman S.R., Breen G.A. (2011). Early dysregulation of the mitochondrial proteome in a mouse model of Alzheimer’s disease. J. Proteomics.

[83] Chandrasekaran K., Hatanpaa K., Rapoport S.I., Brady D.R. (1997). Decreased expression of nuclear and mitochondrial DNA-encoded genes of oxidative phosphorylation in association neocortex in Alzheimer disease. Brain Res. Mol. Brain Res..

[84] Reddy P.H., McWeeney S., Park B.S., Manczak M., Gutala R.V., Partovi D., Jung Y., Yau V., Searles R., Mori M. (2004). Gene expression profiles of transcripts in amyloid precursor protein transgenic mice: Up-regulation of mitochondrial metabolism and apoptotic genes is an early cellular change in Alzheimer’s disease. Hum. Mol. Genet..

[85] Hirai K., Aliev G., Nunomura A., Fujioka H., Russell R.L., Atwood C.S., Johnson A.B., Kress Y., Vinters H.V., Tabaton M. (2001). Mitochondrial abnormalities in Alzheimer’s disease. J. Neurosci..

[86] Hundal T., Norling B., Ernster L. (1984). The oligomycin sensitivity conferring protein (OSCP) of beef heart mitochondria: Studies of its binding to F1 and its function. J. Bioenerg. Biomembr..

[87] Liu F., Shi J., Tanimukai H., Gu J., Gu J., Grundke-Iqbal I., Iqbal K., Gong C.X. (2009). Reduced O-GlcNAcylation links lower brain glucose metabolism and tau pathology in Alzheimer’s disease. Brain.

[88] Hanover J.A., Krause M.W., Love D.C. (2012). Bittersweet memories: Linking metabolism to epigenetics through O-GlcNAcylation. Nat. Rev. Mol. Cell Biol..

[89] Hart G.W., Akimoto Y., Varki A., Cummings R.D., Esko J.D., Freeze H.H., Stanley P., Bertozzi C.R., Hart G.W., Etzler M.E. (2009). The O-GlcNAc Modification. Essentials of Glycobiology.

[90] Rhein V., Song X., Wiesner A., Ittner L.M., Baysang G., Meier F., Ozmen L., Bluethmann H., Drose S., Brandt U. (2009). Amyloid-beta and tau synergistically impair the oxidative phosphorylation system in triple transgenic Alzheimer’s disease mice. Proc. Natl. Acad. Sci. USA.

[91] Parks J.K., Smith T.S., Trimmer P.A., Bennett J.P., Parker W.D. (2001). Neurotoxic Abeta peptides increase oxidative stress in vivo through NMDA-receptor and nitric-oxide-synthase mechanisms, and inhibit complex IV activity and induce a mitochondrial permeability transition in vitro. J. Neurochem..

[92] Terada T., Therriault J., Kang M.S.P., Savard M., Pascoal T.A., Lussier F., Tissot C., Wang Y.T., Benedet A., Matsudaira T. (2021). Mitochondrial complex I abnormalities is associated with tau and clinical symptoms in mild Alzheimer’s disease. Mol. Neurodegener..

[93] David D.C., Hauptmann S., Scherping I., Schuessel K., Keil U., Rizzu P., Ravid R., Drose S., Brandt U., Muller W.E. (2005). Proteomic and functional analyses reveal a mitochondrial dysfunction in P301L tau transgenic mice. J. Biol. Chem..

[94] Casley C.S., Canevari L., Land J.M., Clark J.B., Sharpe M.A. (2002). Beta-amyloid inhibits integrated mitochondrial respiration and key enzyme activities. J. Neurochem..

[95] Parker W.D., Parks J., Filley C.M., Kleinschmidt-DeMasters B.K. (1994). Electron transport chain defects in Alzheimer’s disease brain. Neurology.

[96] Aksenov M.Y., Tucker H.M., Nair P., Aksenova M.V., Butterfield D.A., Estus S., Markesbery W.R. (1999). The expression of several mitochondrial and nuclear genes encoding the subunits of electron transport chain enzyme complexes, cytochrome c oxidase, and NADH dehydrogenase, in different brain regions in Alzheimer’s disease. Neurochem. Res..

[97] Jacobs R.W., Farivar N., Butcher L.L. (1985). Alzheimer dementia and reduced nicotinamide adenine dinucleotide (NADH)-diaphorase activity in senile plaques and the basal forebrain. Neurosci. Lett..

[98] Adav S.S., Park J.E., Sze S.K. (2019). Quantitative profiling brain proteomes revealed mitochondrial dysfunction in Alzheimer’s disease. Mol. Brain.

[99] Li Y., Park J.S., Deng J.H., Bai Y. (2006). Cytochrome c oxidase subunit IV is essential for assembly and respiratory function of the enzyme complex. J. Bioenerg. Biomembr..

[100] Minghetti L. (2004). Cyclooxygenase-2 (COX-2) in inflammatory and degenerative brain diseases. J. Neuropathol. Exp. Neurol..

[101] Cottrell D.A., Borthwick G.M., Johnson M.A., Ince P.G., Turnbull D.M. (2002). The role of cytochrome c oxidase deficient hippocampal neurones in Alzheimer’s disease. Neuropathol. Appl. Neurobiol..

[102] Nagy Z., Esiri M.M., LeGris M., Matthews P.M. (1999). Mitochondrial enzyme expression in the hippocampus in relation to Alzheimer-type pathology. Acta Neuropathol..

[103] Canevari L., Clark J.B., Bates T.E. (1999). beta-Amyloid fragment 25-35 selectively decreases complex IV activity in isolated mitochondria. FEBS Lett..

[104] Chandrasekaran K., Hatanpaa K., Brady D.R., Stoll J., Rapoport S.I. (1998). Downregulation of oxidative phosphorylation in Alzheimer disease: Loss of cytochrome oxidase subunit mRNA in the hippocampus and entorhinal cortex. Brain Res..

[105] Behl C., Davis J.B., Lesley R., Schubert D. (1994). Hydrogen peroxide mediates amyloid beta protein toxicity. Cell.

[106] Wei W., Wang X., Kusiak J.W. (2002). Signaling events in amyloid beta-peptide-induced neuronal death and insulin-like growth factor I protection. J. Biol. Chem..

[107] Pratico D., Uryu K., Leight S., Trojanoswki J.Q., Lee V.M. (2001). Increased lipid peroxidation precedes amyloid plaque formation in an animal model of Alzheimer amyloidosis. J. Neurosci..

[108] Jang J.H., Surh Y.J. (2002). beta-Amyloid induces oxidative DNA damage and cell death through activation of c-Jun N terminal kinase. Ann. N. Y. Acad. Sci..

[109] Tamagno E., Guglielmotto M., Aragno M., Borghi R., Autelli R., Giliberto L., Muraca G., Danni O., Zhu X., Smith M.A. (2008). Oxidative stress activates a positive feedback between the gamma- and beta-secretase cleavages of the beta-amyloid precursor protein. J. Neurochem..

[110] Swerdlow R.H., Khan S.M. (2004). A “mitochondrial cascade hypothesis” for sporadic Alzheimer’s disease. Med. Hypotheses.

[111] Yan M.H., Wang X., Zhu X. (2013). Mitochondrial defects and oxidative stress in Alzheimer disease and Parkinson disease. Free Radic. Biol. Med..

[112] Antonyova V., Kejik Z., Brogyanyi T., Kaplanek R., Pajkova M., Talianova V., Hromadka R., Masarik M., Sykora D., Miksatkova L. (2020). Role of mtDNA disturbances in the pathogenesis of Alzheimer’s and Parkinson’s disease. DNA Repair.

[113] Gredilla R., Bohr V.A., Stevnsner T. (2010). Mitochondrial DNA repair and association with aging—An update. Exp. Gerontol..

[114] Trivedi A., Fantin D.J., Tustanoff E.R. (1986). Role of phospholipid fatty acids on the kinetics of high and low affinity sites of cytochrome c oxidase. Biochem. Cell Biol..

[115] Paradies G., Ruggiero F.M., Petrosillo G., Quagliariello E. (1998). Peroxidative damage to cardiac mitochondria: Cytochrome oxidase and cardiolipin alterations. FEBS Lett..

[116] Richardson J.S. (1993). Free radicals in the genesis of Alzheimer’s disease. Ann. N. Y. Acad. Sci..

[117] Chagnon P., Betard C., Robitaille Y., Cholette A., Gauvreau D. (1995). Distribution of brain cytochrome oxidase activity in various neurodegenerative diseases. Neuroreport.

[118] Kish S.J., Bergeron C., Rajput A., Dozic S., Mastrogiacomo F., Chang L.J., Wilson J.M., DiStefano L.M., Nobrega J.N. (1992). Brain cytochrome oxidase in Alzheimer’s disease. J. Neurochem..

[119] Cadenas E., Boveris A., Ragan C.I., Stoppani A.O. (1977). Production of superoxide radicals and hydrogen peroxide by NADH-ubiquinone reductase and ubiquinol-cytochrome c reductase from beef-heart mitochondria. Arch. Biochem. Biophys..

[120] Ahn B.W., Song D.U., Jung Y.D., Chay K.O., Chung M.A., Yang S.Y., Shin B.A. (2000). Detection of beta-amyloid peptide aggregation using DNA electrophoresis. Anal. Biochem..

[121] Su B., Wang X., Nunomura A., Moreira P.I., Lee H.G., Perry G., Smith M.A., Zhu X. (2008). Oxidative stress signaling in Alzheimer’s disease. Curr. Alzheimer Res..

[122] Korolainen M.A., Goldsteins G., Nyman T.A., Alafuzoff I., Koistinaho J., Pirttila T. (2006). Oxidative modification of proteins in the frontal cortex of Alzheimer’s disease brain. Neurobiol. Aging.

[123] Suthammarak W., Yang Y.Y., Morgan P.G., Sedensky M.M. (2009). Complex I function is defective in complex IV-deficient Caenorhabditis elegans. J. Biol. Chem..

[124] Demarin V., Podobnik S.S., Storga-Tomic D., Kay G. (2004). Treatment of Alzheimer’s disease with stabilized oral nicotinamide adenine dinucleotide: A randomized, double-blind study. Drugs Exp. Clin. Res..

[125] Hou Y., Lautrup S., Cordonnier S., Wang Y., Croteau D.L., Zavala E., Zhang Y., Moritoh K., O’Connell J.F., Baptiste B.A. (2018). NAD(+) supplementation normalizes key Alzheimer’s features and DNA damage responses in a new AD mouse model with introduced DNA repair deficiency. Proc. Natl. Acad. Sci. USA.

[126] Zhang L., Zhang S., Maezawa I., Trushin S., Minhas P., Pinto M., Jin L.W., Prasain K., Nguyen T.D., Yamazaki Y. (2015). Modulation of mitochondrial complex I activity averts cognitive decline in multiple animal models of familial Alzheimer’s Disease. EBioMedicine.

[127] Baumgart M., Priebe S., Groth M., Hartmann N., Menzel U., Pandolfini L., Koch P., Felder M., Ristow M., Englert C. (2016). Longitudinal RNA-Seq Analysis of Vertebrate Aging Identifies Mitochondrial Complex I as a Small-Molecule-Sensitive Modifier of Lifespan. Cell Syst..

[128] Uddin M.S., Kabir M.T., Mamun A.A., Barreto G.E., Rashid M., Perveen A., Ashraf G.M. (2020). Pharmacological approaches to mitigate neuroinflammation in Alzheimer’s disease. Int. Immunopharmacol..

[129] Fiebich B.L., Akter S., Akundi R.S. (2014). The two-hit hypothesis for neuroinflammation: Role of exogenous ATP in modulating inflammation in the brain. Front. Cell. Neurosci..

[130] Martinez J., Marmisolle I., Tarallo D., Quijano C. (2020). Mitochondrial Bioenergetics and Dynamics in Secretion Processes. Front. Endocrinol..

[131] Dixit S., Fessel J.P., Harrison F.E. (2017). Mitochondrial dysfunction in the APP/PSEN1 mouse model of Alzheimer’s disease and a novel protective role for ascorbate. Free Radic. Biol. Med..

[132] Baumgartner H.K., Gerasimenko J.V., Thorne C., Ferdek P., Pozzan T., Tepikin A.V., Petersen O.H., Sutton R., Watson A.J., Gerasimenko O.V. (2009). Calcium elevation in mitochondria is the main Ca2+ requirement for mitochondrial permeability transition pore (mPTP) opening. J. Biol. Chem..

[133] Rizzuto R., Brini M., Murgia M., Pozzan T. (1993). Microdomains with high Ca2+ close to IP3-sensitive channels that are sensed by neighboring mitochondria. Science.

[134] Rizzuto R., Simpson A.W., Brini M., Pozzan T. (1992). Rapid changes of mitochondrial Ca2+ revealed by specifically targeted recombinant aequorin. Nature.

[135] Montero M., Alonso M.T., Carnicero E., Cuchillo-Ibanez I., Albillos A., Garcia A.G., Garcia-Sancho J., Alvarez J. (2000). Chromaffin-cell stimulation triggers fast millimolar mitochondrial Ca2+ transients that modulate secretion. Nat. Cell Biol..

[136] McCormack J.G., Halestrap A.P., Denton R.M. (1990). Role of calcium ions in regulation of mammalian intramitochondrial metabolism. Physiol. Rev..

[137] Solesio M.E., Garcia Del Molino L.C., Elustondo P.A., Diao C., Chang J.C., Pavlov E.V. (2020). Inorganic polyphosphate is required for sustained free mitochondrial calcium elevation, following calcium uptake. Cell Calcium.

[138] Solesio M.E., Demirkhanyan L., Zakharian E., Pavlov E.V. (2016). Contribution of inorganic polyphosphate towards regulation of mitochondrial free calcium. Biochim. Biophys. Acta.

[139] Solesio M.E., Elustondo P.A., Zakharian E., Pavlov E.V. (2016). Inorganic polyphosphate (polyP) as an activator and structural component of the mitochondrial permeability transition pore. Biochem. Soc. Trans..

[140] Khong M.L., Li L., Solesio M.E., Pavlov E.V., Tanner J.A. (2020). Inorganic polyphosphate controls cyclophilin B-mediated collagen folding in osteoblast-like cells. FEBS J..

[141] Sheehan J.P., Swerdlow R.H., Miller S.W., Davis R.E., Parks J.K., Parker W.D., Tuttle J.B. (1997). Calcium homeostasis and reactive oxygen species production in cells transformed by mitochondria from individuals with sporadic Alzheimer’s disease. J. Neurosci..

[142] Yu W., Jin H., Huang Y. (2021). Mitochondria-associated membranes (MAMs): A potential therapeutic target for treating Alzheimer’s disease. Clin. Sci..

[143] Calvo-Rodriguez M., Hou S.S., Snyder A.C., Kharitonova E.K., Russ A.N., Das S., Fan Z., Muzikansky A., Garcia-Alloza M., Serrano-Pozo A. (2020). Increased mitochondrial calcium levels associated with neuronal death in a mouse model of Alzheimer’s disease. Nat. Commun..

[144] Jaworska A., Dzbek J., Styczynska M., Kuznicki J. (2013). Analysis of calcium homeostasis in fresh lymphocytes from patients with sporadic Alzheimer’s disease or mild cognitive impairment. Biochim. Biophys. Acta.

[145] Hedskog L., Pinho C.M., Filadi R., Ronnback A., Hertwig L., Wiehager B., Larssen P., Gellhaar S., Sandebring A., Westerlund M. (2013). Modulation of the endoplasmic reticulum-mitochondria interface in Alzheimer’s disease and related models. Proc. Natl. Acad. Sci. USA.

[146] Boyman L., Greiser M., Lederer W.J. (2021). Calcium influx through the mitochondrial calcium uniporter holocomplex, MCUcx. J. Mol. Cell Cardiol..

[147] Perez M.J., Ponce D.P., Aranguiz A., Behrens M.I., Quintanilla R.A. (2018). Mitochondrial permeability transition pore contributes to mitochondrial dysfunction in fibroblasts of patients with sporadic Alzheimer’s disease. Redox. Biol..

[148] Patergnani S., Suski J.M., Agnoletto C., Bononi A., Bonora M., De Marchi E., Giorgi C., Marchi S., Missiroli S., Poletti F. (2011). Calcium signaling around Mitochondria Associated Membranes (MAMs). Cell Commun. Signal..

[149] Area-Gomez E., Del Carmen Lara Castillo M., Tambini M.D., Guardia-Laguarta C., de Groof A.J., Madra M., Ikenouchi J., Umeda M., Bird T.D., Sturley S.L. (2012). Upregulated function of mitochondria-associated ER membranes in Alzheimer disease. EMBO J..

[150] Supnet C., Bezprozvanny I. (2010). The dysregulation of intracellular calcium in Alzheimer disease. Cell Calcium.

[151] Jones K.T., Sharpe G.R. (1994). Thapsigargin raises intracellular free calcium levels in human keratinocytes and inhibits the coordinated expression of differentiation markers. Exp. Cell Res..

[152] Deniaud A., Sharaf el dein O., Maillier E., Poncet D., Kroemer G., Lemaire C., Brenner C. (2008). Endoplasmic reticulum stress induces calcium-dependent permeability transition, mitochondrial outer membrane permeabilization and apoptosis. Oncogene.

[153] Gibson G.E., Zhang H., Toral-Barza L., Szolosi S., Tofel-Grehl B. (1996). Calcium stores in cultured fibroblasts and their changes with Alzheimer’s disease. Biochim. Biophys. Acta.

